# Characteristics and relationship between hyperphagia, anxiety, behavioral challenges and caregiver burden in Prader-Willi syndrome

**DOI:** 10.1371/journal.pone.0248739

**Published:** 2021-03-25

**Authors:** Nathalie Kayadjanian, Caroline Vrana-Diaz, Jessica Bohonowych, Theresa V. Strong, Josée Morin, Diane Potvin, Lauren Schwartz

**Affiliations:** 1 Foundation for Prader-Willi Research, Walnut, California, United States of America; 2 PWS-Clinical Trial Consortium, Walnut, California, United States of America; 3 Department of Genetics, University of Alabama, Birmingham, Alabama, United States of America; 4 Excelsus Statistics, Montreal, Quebec, Canada; 5 Department of Rehabilitation Medicine, University of Washington, Seattle, Washington, United States of America; University College London, UNITED KINGDOM

## Abstract

**Objectives:**

Prader-Willi syndrome (PWS) is a rare genetic disorder characterized by maladaptive behaviors, amongst which hyperphagia is a life-long concern for individuals with PWS and their caregivers. The current study examined the contribution of hyperphagia and other factors to caregiver burden across lifespan, in 204 caregivers of individuals with PWS living in the US, using the Zarit Burden Interview (ZBI) and the hyperphagia questionnaire (HQ-CT).

**Results:**

We found a strong relationship between ZBI and HQ-CT especially in individuals with PWS older than 4 y and showed that HQ-CT scores of individuals with PWS is positively correlated with ZBI scores of their caregivers. The weight status of individuals with PWS was not associated with HQ-CT and ZBI scores, except for obese individuals who had significantly higher HQ-CT scores when compared to normal weight PWS individuals. We looked at PWS symptoms and care-related issues that impacted individuals and caregivers the most. We found that care-related tasks had the biggest negative impact on caregivers of children aged 0–4 y, whereas anxiety, temper tantrums, and oppositional behaviors of older individuals with PWS had the biggest impact on their caregivers concomitant with their high caregiver burden. Finally, we assessed the variability of HQ-CT and ZBI over 6 months in a subgroup of 83 participants. Overall, neither measure differed between 6 months and baseline. Most individual’s absolute HQ-CT score changes were between 0–2 units, whereas absolute ZBI score changes were between 0–6 points. Changes in the caregiver’s or individual’s life had little or no effect on HQ-CT and ZBI scores.

**Conclusions:**

This study demonstrates a relationship between hyperphagia and caregiver burden and sheds light on predominant symptoms in children and adolescents that likely underly PWS caregiver burden. The stability and relationship between HQ-CT and ZBI support ZBI as an additional outcome measure in PWS clinical trials.

## Introduction

Prader-Willi syndrome (PWS) is a rare neurodevelopmental disorder that occurs in approximately one in every 15,000 to 30,000 births [1]. It is caused by a lack of expression of paternally inherited imprinted genes on chromosome 15q11-q13 and affects all races and ethnicities, males and females with equal frequency. PWS is a multisystem disorder and its manifestations are complex with changing clinical features across the individual’s lifespan that incur high caregiver burden.

Individuals with PWS present with central hypotonia and feeding problems in early infancy, followed during childhood and adulthood by intellectual and learning disabilities, maladaptive behaviors, hypogonadism and incomplete sexual development, short stature due to growth hormone deficiency and severe hyperphagia, which is a hallmark symptom of the syndrome. Emotional and behavioral challenges are prominent in PWS and include anxiety, repetitive behaviors, temper outbursts, and oppositional behavior [2–7]. As individuals with PWS reach adulthood, they are at high risk for developing psychiatric illness, including psychosis and major depression [8]. Growth hormone therapy is the only FDA-approved therapy for use in children with PWS. Despite beneficial effects on height, body composition, strength, endurance, bone mineral density, respiratory quotient, and sense of well-being [9, 10] it has no discernable impact on hyperphagia or behavioral challenges. The severe and life-long hyperphagia, behavioral symptoms and lack of treatments all create unique challenges in caring for persons with PWS.

Family caregivers of individuals with PWS provide considerable support to their children and, in turn, experience significant burden [11–13]. Siblings of individuals with PWS may also experience significant stress and anxiety [14, 15]. In a previous study [16], we showed that caregiver burden as measured by the Zarit Burden Interview (ZBI) is strikingly high in PWS. We also found significant negative impacts of PWS on caregiver work, sleep, romantic relationships and emotional well-being, and showed that ZBI is a good predictor of these negative impacts. The levels of caregiver burden in PWS were similar to those measured in caregivers of individuals with autism spectrum disorder [17, 18] but higher than those measured in caregivers for persons with dementia, Alzheimer’s disease, and traumatic brain injury [19–21]. The high levels of caregiver burden found in PWS likely reflect the challenges and difficulties caring for a child with a life-long neurodevelopmental condition but also the complexity of this particular syndrome.

Our previous study showed that the level of caregiver burden increases with the age of the individuals with PWS, reaching its highest levels in caregivers of adolescents and young adults [16]. This could reflect both the changes over time of the clinical manifestations and severity of symptom characteristics of individuals with PWS. The age of onset for hyperphagia in PWS varies, starting as early as the age of 3, with the average onset at 8 years of age [22]. Hyperphagia causes intense food cravings and food seeking that result in uncontrollable weight gain and can lead to morbid obesity. Even under strictly controlled food environment and diet, the person with PWS is at increased risks of death caused by choking or gastric perforations after consuming more food than usual [23]. The persistent drive for food and continuous supervision required by caregivers impact the quality of life of individuals with PWS and likely contribute to significant caregiver burden in PWS. In addition to hyperphagia, emotional, behavioral and psychiatric problems are more pronounced during adolescence and young adulthood [3, 5, 24–27]. These challenges can be severe and negatively impact the individual’s schooling, work opportunities and family well-being [28]. However, no studies have previously assessed the degree to which hyperphagia, emotional, and behavioral symptoms in PWS and issues related to caring for an individual with PWS may contribute to caregiver burden.

Measuring hyperphagia has long been a challenge [29]. Weight gain has often been used as a proxy-measure of hyperphagia in obese individuals with PWS. However, in individuals with PWS who are living under strict dietary requirements, weight is tightly controlled and may not reflect the degree of hyperphagia in these individuals. The hyperphagia questionnaire developed by Dykens and collaborators in 2007 [29] has paved the way for the development of the hyperphagia questionnaire for use in clinical trials (HQ-CT). HQ-CT is a validated caregiver-reported measure of food-seeking behaviors observed among individuals with PWS that has incorporated industry guidance related to clinical outcome assessment and FDA recommendations [30]. Among the four ongoing phase 3 clinical trials for hyperphagia in PWS, three trials are using HQ-CT as their primary outcome measure (NCT03440814, NCT03649477, NCT03790865). Little is known, however, about the natural variation of HQ-CT scores across ages and stability over time. Additionally, little is known about the impact of hyperphagia on the burden of caring for the person with PWS.

The present study was conducted to extend the findings from our initial study on caregiver burden in PWS [16], to further explore the relationship between hyperphagia and caregiver burden, and to evaluate the contribution of other factors to caregiver burden in PWS. It is the first study to explore the variability of hyperphagia across ages and stability over time. Specifically, we concomitantly measured HQ-CT scores (as a measure of hyperphagia) in individuals with PWS across different ages and ZBI scores (as a measure of caregiver burden) in their caregivers, and analyzed their relationship. We also measured body mass index (BMI) and HQ-CT scores in individuals with PWS to examine the relationship between the degree of hyperphagia and weight status. To better understand the factors contributing to caregiver burden in PWS, we looked at the PWS symptoms and issues related to caregiving that had the biggest negative impact on individuals with PWS and their caregivers across different age groups. Finally, we measured HQ-CT and ZBI changes over two time points separated by 6 months to assess the natural variability of these measures over a time that often represents the duration of a phase 3 clinical trial in the PWS population.

## Methods

### Participants

Participants recruited through US PWS advocacy groups were asked to complete two online surveys at two time points separated by 6 months in the Global PWS registry [31]. Two hundred and four respondents who identified the United States as their country of residence were included in this study. At 6 months, 83 out of the 204 initial responders completed a second survey.

### Assessment

The baseline survey, comprised of 46 questions, included questions on demographics of individuals with PWS and their caregivers, HQ-CT, ZBI, and additional questions related to the impact of PWS specific symptoms and care related issues on individuals with PWS and their caregivers. The survey at 6 months was comprised of the same 46 baseline questions and 2 additional questions on whether the person with PWS and/or the caregiver experienced a significant life event or changes in health or behavior in the last 6 months. If the person with PWS did have a significant change, caregivers specified whether it was related to a change in living situation of the person with PWS, change in health of the person with PWS, treatment for mood or behavior, treatment for hunger or other significant change. If the caregiver had a significant change, the caregiver specified whether change was related to change in their living situation, their health status or other caregiver-related change.

#### HQ-CT

Hyperphagia-related behaviors were assessed by the HQ-CT, a caregiver-reported instrument composed of 9 items designed to measure food-related behaviors in PWS utilizing a 2-week recall period. Responses to each item range from 0 to 4 units and possible total scores range from 0 (no hyperphagia) to 36 (high degree of hyperphagia). HQ-CT has been shown to have good internal consistency, content validity, and test-retest reliability [30].

#### ZBI

We used the 22-item self-report ZBI questionnaire (Copyright 1980, 1983, 1990 Steven H Zarit and Judy M Zarit) as described [16]. Briefly, ZBI measures caregiver subjective burden in health, psychological well-being, finances, social life and relationship with the patient. For each question, caregivers rated their experiences on a 5-point Likert scale where 0  =  never and 4  =  nearly always. Responses were used to derive the ZBI total score with possible total scores range from 0 (no burden) to 88 (highest burden).

#### Impact of PWS symptoms and caregiver-related issues

As part of the online survey, caregivers were asked to choose 3 symptoms amongst a list of 12 (S1 Appendix) that had the biggest negative impact on the person with PWS. Caregivers were asked to rate each of these symptoms on a scale from 0 to 9 where 0 = not at all a challenge to 9 = extreme challenge. Level of intensity was used as a quality control measure for the importance of the symptom chosen by participants. All responses were included in the analyses including caregivers who selected less than 3 symptoms (2.9%) and those who selected more than 3 symptoms (37.2%). Caregivers were next asked to choose 3 issues amongst a list of 14 symptoms and care related issues that had the greatest impact on them and to indicate the level of severity on a scale from 0 (not a challenge) to 9 (extremely challenging). All responses were included in the analyses including caregivers who selected less than 3 symptoms (1.5%) and those who selected more than 3 symptoms (33.3%).

#### Weight status

The date of birth was asked to participants in addition to their age category. Weight, height, and age-derived from date of birth of individuals with PWS were reported by caregivers and used to calculate the BMI by dividing the weight in kilograms by the square of the height in meters. The interpolated weight and height data were checked for biologically implausible values, which were removed from this analysis. For individuals with PWS aged between 2 y and below 20 y, weight status was defined based on BMI-for-age and sex percentiles calculated from the CDC growth charts (https://www.cdc.gov/nccdphp/dnpao/growthcharts/ resources/sas.htm) with the corresponding percentile range: underweight–below 5^th^, normal weight–between 5 ^th^ -85^th^, overweight–between 85^th^-95^th^, and obese–between 95^th^-100^th^ percentile. For adults with PWS aged 20 years and older, the commonly accepted BMI ranges were used: underweight–less than 18.5 kg/m^2^, normal weight–between 18.5 and 24.9 kg/m^2^, overweight–between 25.0 and 29.9 kg/m^2^, and obese–BMI ≥ 30 kg/m^2^.

### Procedures and ethical statement

The survey questionnaires were implemented in the web-based Global PWS registry [31]. The Global PWS Registry is compliant with US Health Information Privacy Laws, FDA regulations on electronic records, and the security requirements of the European Union General Data Protection Regulation. Registry data is only accessed by registry study personnel. Only de-identified data were analyzed in this study as per the Registry protocol. The survey questionnaires and recruitment materials were reviewed and approved by the Hummingbird IRB committee (#2017-57-FPWR).

### Statistical analyses

Descriptive statistics are given in number, proportion, mean, standard deviation (SD), and median. One-way analysis of variance (ANOVA) was conducted to compare the association of individual’s age group and ZBI or HQ-CT, the association of individual’s weight status and ZBI or HQ-CT. When the association was found significant, post hoc comparisons were performed using the Tukey`s adjustment. Spearman correlation coefficient was used to assess the strength of association between HQ-CT and ZBI scores. A linear regression model was used to assess whether HQ-CT scores predicted ZBI scores and whether the weight status of individuals with PWS predicted HQ-CT scores. Cochran Mantel-Haenzel tests were used to compare the proportion of participants who selected a given symptom or issue, stratified by age categories. A paired t-test was used to compare HQ-CT or ZBI scores at baseline and at 6 months or to compare HQ-CT or ZBI score changes from baseline at 6 months in function of life or event changes. The effect of age groups on HQ-CT or ZBI scores across the two time-points involved an ANOVA test using a model including age group, time (baseline or 6 month) with an unstructured variance-covariance matrix to model the within-subject correlation. HQ-CT or ZBI score comparisons between age groups across the two time-points were assessed using least square means difference using Tukey`s adjustments. P-values < 0.05 were used for statistical significance. All analyses were performed using SAS 9.4 (SAS Institute, Cary, NC) version 9.4.

## Results

### Participants

#### Caregivers

Out of the 204 study participants living in the United States, 99% were primary caregivers. For simplicity, all participants were labelled “caregivers” in this study. Caregivers were predominantly composed of mothers (94%), aged between 30 and 59 years (91%), married or in a relationship (87%) and Caucasian non-Hispanic whites (90%) (Table 1). More than 55% of caregivers reported an annual household income equal or greater than $75,000 per year including 50% having an income greater or equal to $100,000 yearly (Table 1). Household were predominantly composed of 4 members or less (Table 1).

**Table 1:** Sociodemographic characteristics of participating PWS caregivers and individuals with PWS living in the US.

Variable	N (%)	Variable	N (%)
Caregivers			
Relationship with Child		Annual household income	
Parent	201 (98.53)	Less than $10000	7 (3.43)
Grandparent	1 (0.49)	$10000 - $14999	11 (5.39)
Sibling	1 (0.49)	$15000 - $19999	6 (2.94)
Legal guardian	1 (0.49)	$20000 - $24999	1 (0.49)
Age (years)		$25000 - $29999	3 (1.47)
20–29	10 (4.90)	$30000 - $34999	7 (3.43)
30–39	64 (31.37)	$35000 - $39999	3 (1.47)
40–49	74 (36.27)	$40000 - $44999	6 (2.94)
50–59	47 (23.04)	$45000 - $49999	8 (3.92)
60–69	7 (3.43)	$50000 - $54999	5 (2.45)
70–79	2 (0.98)	$55000 - $59999	8 (3.92)
Marital status		$60000 - $74999	17 (8.33)
Married	171 (83.82)	$75000 - $84999	16 (7.84)
Living with partner	6 (2.94)	$85000 - $99999	5 (2.45)
Divorced	11 (5.39)	$100000 - $149999	54 (26.47)
Separated	8 (3.92)	$150000 - $199999	14 (6.86)
Widow	2 (0.98)	$200000 - $249999	10 (4.90)
Single	6 (2.94)	$250000 and above	14 (6.86)
Ethnicity		Don’t know	5 (2.45)
Asian	4 (1.96)	Decline to provide	4 (1.96)
Caucasian/ White non-Hispanic	183 (89.71)	**Individuals with PWS**	
Black / African American	4 (1.96)	Age (years)	
Hispanic/ Latino	7 (3.43)	0–4	53 (25.98)
American Indian/Alaska Native	1 (0.49)	5–11	70 (34.31)
Multi-ethnic	3 (1.47)	12–18	44 (21.57)
Other	1 (0.49)	19–30	33 (16.18)
Decline to answer	1 (0.49)	31+	4 (1.96)
Household		Living situations	
2	13 (6.37)	One or both parents	196 (96.08)
3	48 (23.53)	Residential/Boarding school	2 (0.98)
4	74 (36.27)	Independent apartment/house with supports	2 (0.98)
5	42 (20.59)	Small supported living (4 or less)	1 (0.49)
6	20 (9.8)	Group home (5 or more)	1 (0.49)
7	5 (2.45)	Other	2 (0.98)
8	1 (0.49)	Time living with caregivers	
9	1 (0.49)	0–25%	5 (2.45)
		26–50%	1 (0.49)
		51–75%	3 (1.47)
		76–100%	195 (95.59)

#### Individuals with PWS

The age of the individuals with PWS was relatively well distributed across infants to early adulthood, except for individuals aged 31 years and above who were comparatively under-represented (Table 1). Most individuals with PWS (96%) lived with one or both parents and spent the majority of their time with their caregivers (Table 1).

### Characteristics and relationship between hyperphagia and caregiver burden across ages

#### HQ-CT

**Fig 1:**
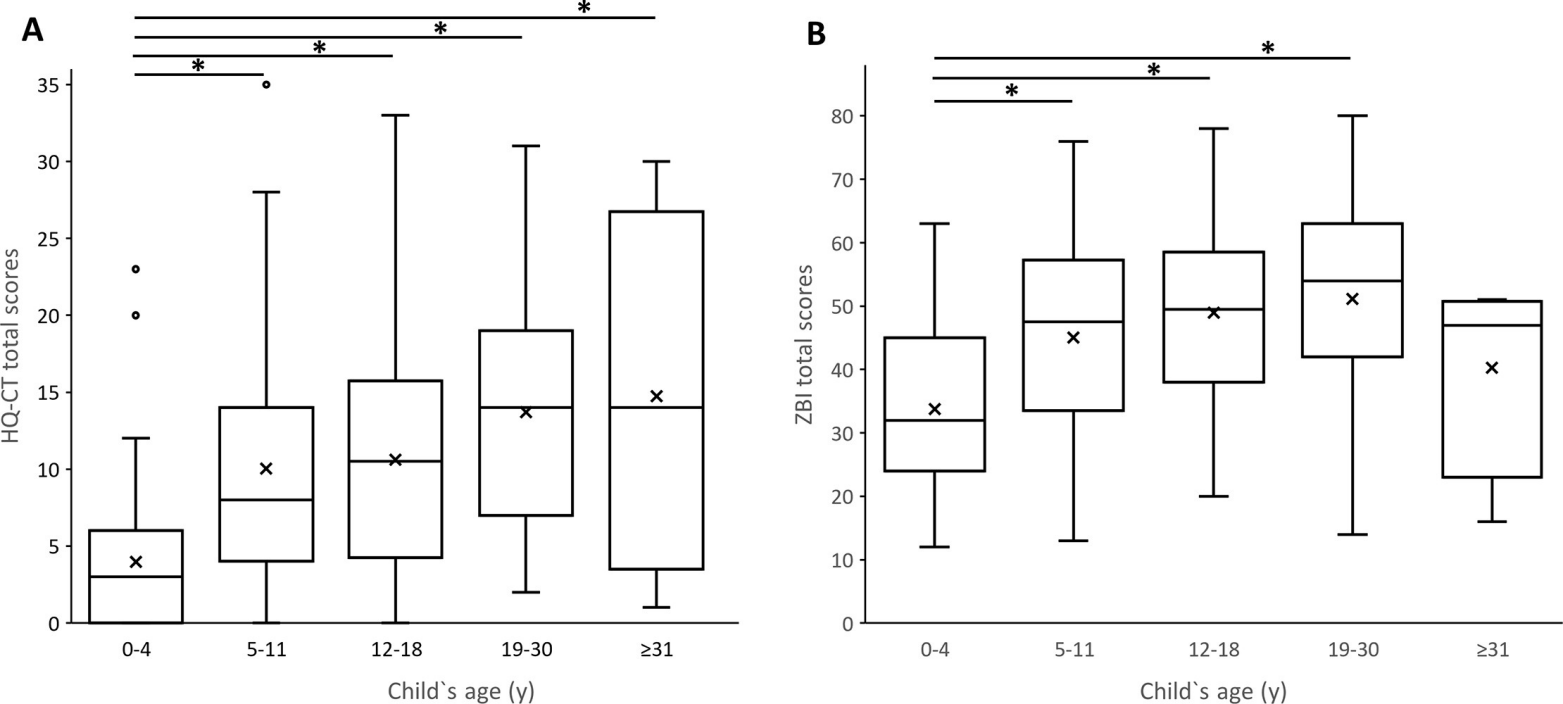
HQ-CT total scores (A) and ZBI total scores (B) vary with the age of individuals with PWS. Box-and-whisker plots showed the range of HQ-CT total scores (left) and ZBI total scores (right) in function of individual’s age groups (*p < 0.05). Within the boxplot, the mean value is represented by the black cross, the median by the horizontal dividing line, and the top and bottom of the box represent the seventy-fifth and twenty-fifth percentile, with the whiskers indicating the maximum and minimum points and outlier points shown as small empty circles.

The mean (SD) baseline HQ-CT score for all 204 individuals with PWS was 9.27 (8.13) with a median score of 7 and scores ranging from 0 to 35. HQ-CT scores varied significantly across the 5-age groups of individuals with PWS (F (4, 199) = 10.68, p < 0.0001). Post hoc comparisons using the Tukey`adjustments indicated that the HQ-CT scores (mean (SD)) in the 5–11 y (10.02 (8.24)), 12–18 y (10.61 (8.06)), 19–30 y (13.69 (7.72)), and in adults aged 31 y or more (14.75 (12.12)) categories were significantly higher than HQ-CT scores for children with PWS in the 0–4 y age category (3.98 (4.8)) (p < 0.05 when compared to 0–4 y category) (Fig 1A). Although HQ-CT scores tended to increase with the child’s age, no statistically significant score differences were found between 5–11 y, 12–18 y, 19–30 y and adults aged 31 years and above (p > 0.05). The HQ-CT scores in the oldest age group were spread out over a larger range of values (Fig 1A). It is noteworthy that some children aged between 0 and 11 y had very high HQ-CT scores (Fig 1A) reflecting a high degree of hyperphagia at an early age. Overall, these results suggest that HQ-CT scores are significantly lower in children aged 4 y and below. For older children, HQ-CT scores tend to increase with age albeit with an increased range of HQ-CT scores.

#### Zarit Burden Interview (ZBI)

The mean (SD) baseline ZBI score for all 204 caregivers was 43.86 (15.87) with a median score of 45 and scores ranging from 12 to 80. Caregiver burden varied significantly across the 5-age group of individuals with PWS (F (4,199) = 9.86, p < 0.0001) (Fig 1B). Post hoc comparisons using the Tukey’s adjustments indicated that the ZBI total score (mean (SD)) for caregivers of individuals in the 5–11 y (45.07 (14.99)), 12–18 y (48.96 (14.51)) and 19–30 y (51.15 (16.76)) categories were significantly higher than in the 0–4 y age category (33.76 (12.67)) (p < 0.05 when compared to 0–4 y group). Although ZBI scores tended to increase with the child’s age, no statistically significant score changes were found between 5–11 y, 12–18 y and 19–30 y age categories. The ZBI total score of caregivers of adults aged 31 y or more (40.25 (16.46)) was not statistically different from the 0–4 y age category (p > 0.05). Taken together, these results suggest that the level of caregiver burden was the lowest for caregivers of infants and young children below 4 y of age. Level of burden increased in caregivers of children aged 5 y and above and reached its highest levels in caregivers of young adults aged between 19–30 y. Caregiver burden tended to decrease in the caregivers of the four adults aged 31 y and older to levels similar to those caring for the youngest PWS group.

#### Relationship between HQ-CT and ZBI

**Fig 2:**
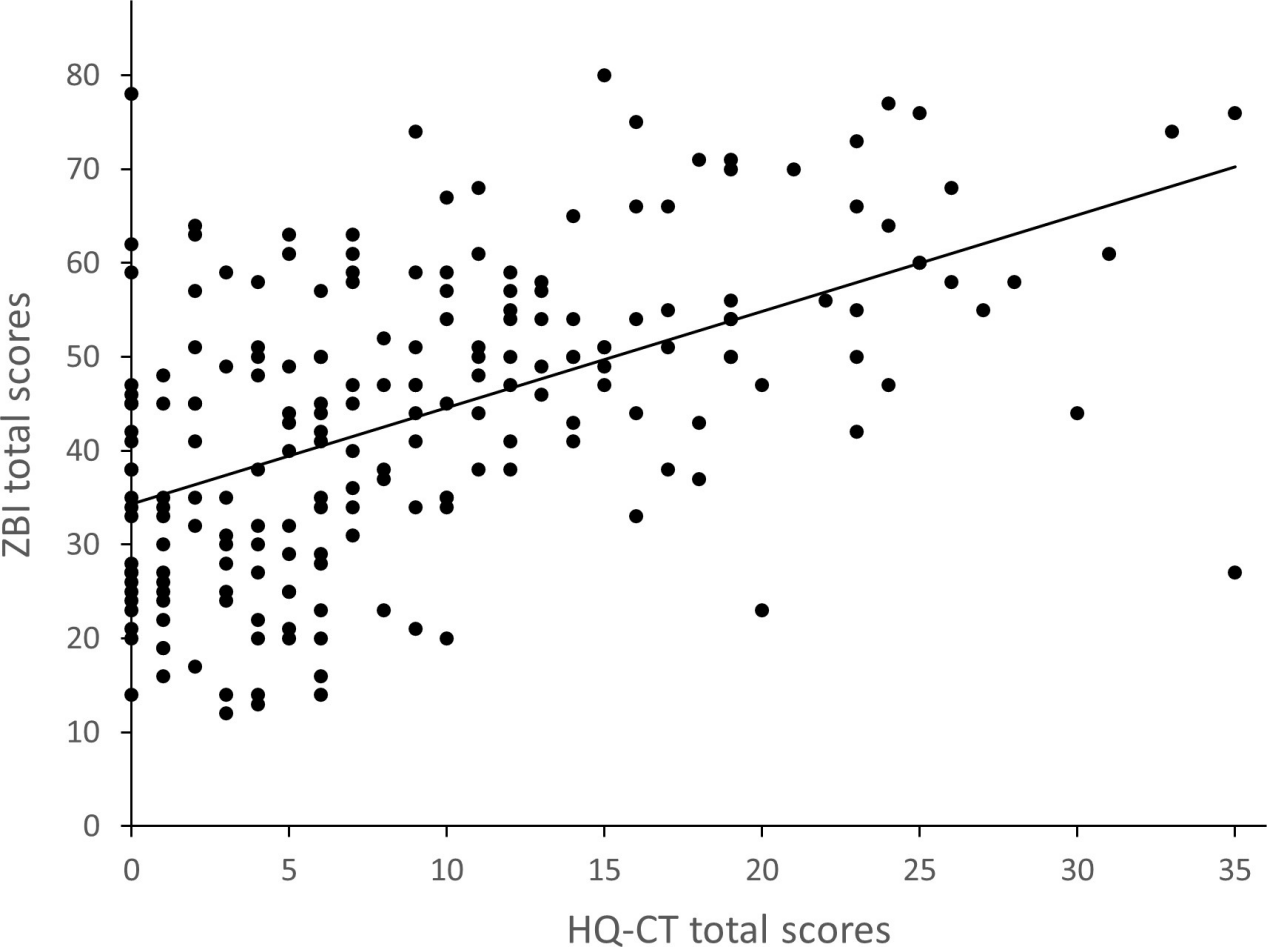
Relationship between hyperphagia and caregiver burden. Shows HQ-CT and ZBI total scores for each of the 204 participants of the study.

Globally, HQ-CT total scores correlated strongly, positively and significantly with ZBI total scores (Spearman coefficient of correlation (r = 0.53, p *<* 0.001) (Fig 2). HQ-CT scores of individuals with PWS within the 5–11 y, 12–18 y and 19–30 y age categories, were strongly and significantly correlated with ZBI scores (r = 0.47, p < 0.0001, r = 0.51, p < 0.001, r = 0.50, p < 0.01, respectively). In contrast, HQ-CT scores of infants and children aged between 0–4 y were not correlated with ZBI scores (r = 0.12, p > 0.05). Although HQ-CT scores of adults aged 31 y and older were moderately correlated with ZBI scores (r = 0.40), they did not reach statistical significance (p > 0.05).

A linear regression model was used to assess if HQ-CT scores of individuals with PWS (with age as a covariate) predicted caregiver burden. A significant regression equation was found (F (5,198) = 19.2, p < 0.0001) with a coefficient of determination (R^2^) of 0.33. Overall, for every 1-point increase in HQ-CT score, a 0.87 increase in ZBI score was found (parameter estimate = 0.87). Although we found no interaction between age groups and HQ-CT (p > 0.1), age was a significant covariate in this generalized linear model (F (4) = 3.92, p < 0.01). The intercept (estimated mean ZBI scores when HQ-CT scores equal to zero) for caregivers of individuals aged 0–4 y was 30.3. When compared to the 0–4 y age group, the intercept was significantly higher for caregivers of individuals aged 5–11 y (estimated mean = 36.4, p < 0.05), 12–18 y (estimated mean = 39.8, p < 0.001), and 19–30 y (estimated mean = 39.3, p < 0.01), while it was not statistically different for caregivers of individuals aged 31 y and older (estimated mean = 27.5, p > 0.05).

Altogether, these results showed that HQ-CT scores of individuals with PWS are associated with ZBI scores of their caregivers.

### Relationship between HQ-CT, ZBI and weight status

The removal of data of individuals from this analysis due to missing age allowing to derive BMI-for-age percentile (n = 17), biologically implausible values (BIV) (n = 8) or age below 2 y (n = 10) did not affect the age distribution (Table 2) which was similar to the distribution of the total population (compare to Table 1). Overall, 43% of individuals with PWS in our study were in the normal weight range with most individuals aged 18 y or less (Table 2). Only 5% of individuals with PWS were underweight. The overweight and obese subpopulation represented 53% of the individuals with PWS. Obese individuals accounted for 32.5% with most aged 19 y and older. It is noteworthy that obese individuals with PWS were found in all age categories including young children (Table 2).

**Table 2:** Relationship between HQ-CT and weight status across age groups.

Age group (year)	2–4		5–11		12–18		19–30		≥31		All ages	
Weight status	N	%	N	%	N	%	N	%	N	%	N	%
Underweight	3	1.78	1	0.59	4	2.37					8	4.73
Normal Weight	17	10.06	31	18.3	15	8.88	8	4.73	1	0.59	72	42.6
Overweight	5	2.96	14	8.28	7	4.14	8	4.73			34	20.12
Obese	12	7.1	16	9.47	9	5.33	15	8.88	3	1.78	55	32.54

N represents to number of individuals per weight status and age group categories. The % represents the proportion of individuals with PWS relative to the total number of participants (n = 169).

**Fig 3:**
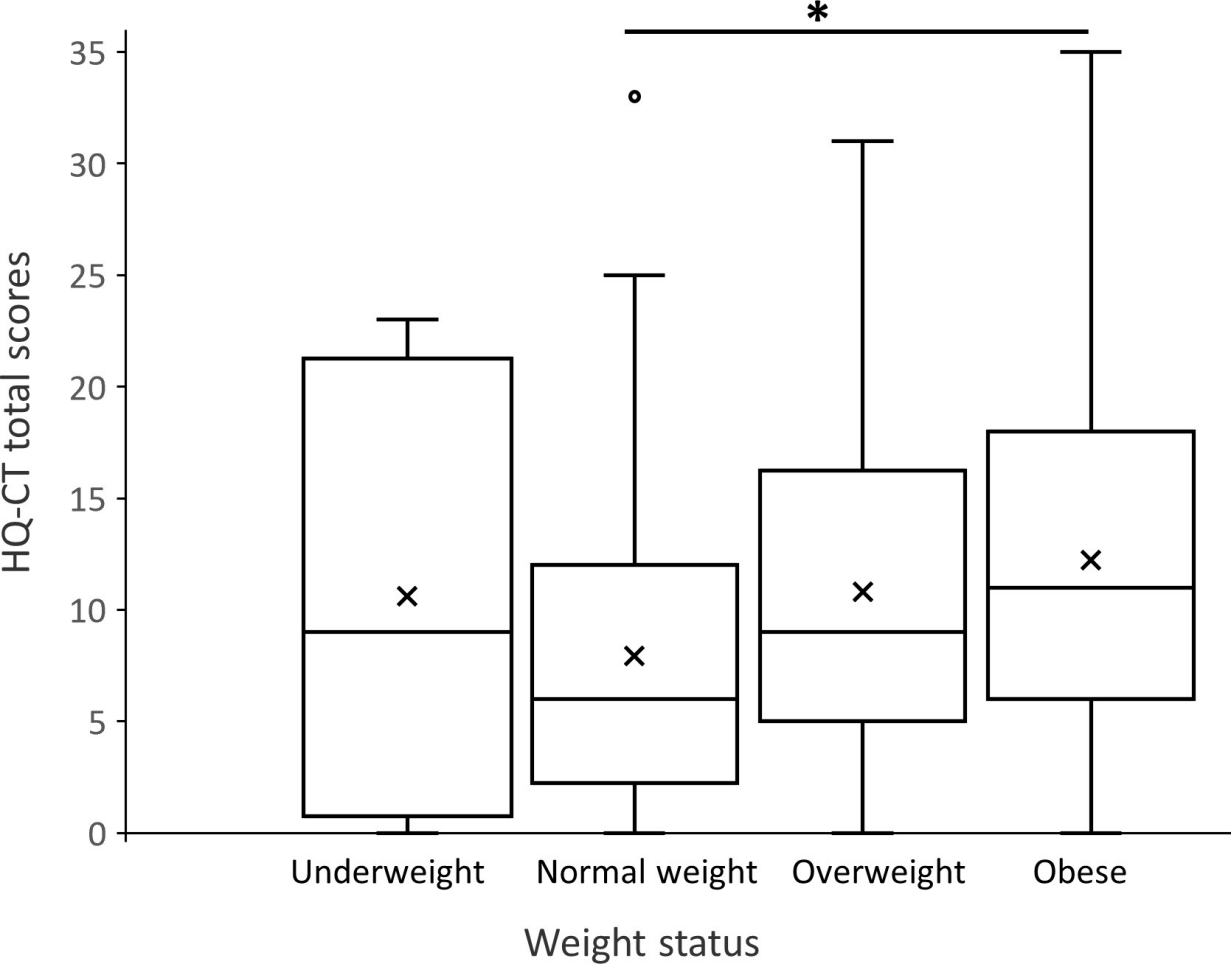
Relationship between weight status and hyperphagia. Box-and-whisker plots showed the range of HQ-CT total scores in function of individual’s weight status (*p < 0.05). Within the boxplot, the mean value is represented by the black cross, the median by the horizontal dividing line, and the top and bottom of the box represent the seventy-fifth and twenty-fifth percentile, with the whiskers indicating the maximum and minimum points and outlier points shown as small empty circles.

Globally, the HQ-CT total scores varied significantly with the weight status of individuals with PWS (F (3,165) = 3.29, p < 0.05) (Fig 3). Pairwise comparisons between mean HQ-CT scores and weight status using the Tukey’s adjustment indicated that HQ-CT scores were significantly higher in obese individuals compared to individuals with normal weight (p < 0.05), but were not different from scores of underweight and overweight individuals with PWS (p > 0.05) (Fig 3). The mean HQ-CT scores did not significantly differ between normal weight, underweight and overweight individuals (p > 0.05). It is noteworthy that HQ-CT scores in underweight individuals were spread out over a larger range of values than in normal weight, overweight and obese individuals with PWS (Fig 3).

A linear regression model was used to assess if the weight status of individuals with PWS (with age group as a covariate) predicted HQ-CT scores. We found no interaction between age group and weight status (p > 0.1). A significant regression equation was found (F (7,161) = 5.1, p < 0.0001) with a R^2^ value of 0.18. However, weight status did not significantly predict HQ-CT scores (F (3) = 2.46, p > 0.05). From this model, the mean HQ-CT score for underweight and overweight individuals was not statistically different from the mean HQ-CT score of individuals with normal weight (p > 0.1). In contrast, obese individuals with PWS had a significantly higher mean HQ-CT score when compared to individuals with normal weight (mean HQ-CT score = 7.07 compared to 3.54, p = 0.01). The age of the individuals with PWS was a significant covariate (F (4) = 6.15, p = 0.0001), in that the mean HQ-CT score increased significantly with the age group of individuals with PWS. Altogether, this model indicates that the age of the individual, not weight status, is significantly correlated with HQ-CT scores.

ZBI total scores were not related to the weight status of individuals with PWS (F (3,165) = 1.36, p > 0.05). The ZBI total scores (mean (SD)) for caregivers of underweight, normal weight, overweight and obese individuals with PWS were: 46.75 (20.21), 41.67 (15.31), 47.5 (15.32), 46.05 (16.90), respectively.

Taken together, our results suggest that the weight status of individuals with PWS is not predictive of their degree of hyperphagia except for obese individuals who have higher hyperphagia scores than normal weight individuals. In addition, our results indicate that caregiver burden is not related to the weight status of individuals with PWS.

### PWS symptoms that have the biggest impact on individuals with PWS

**Fig 4:**
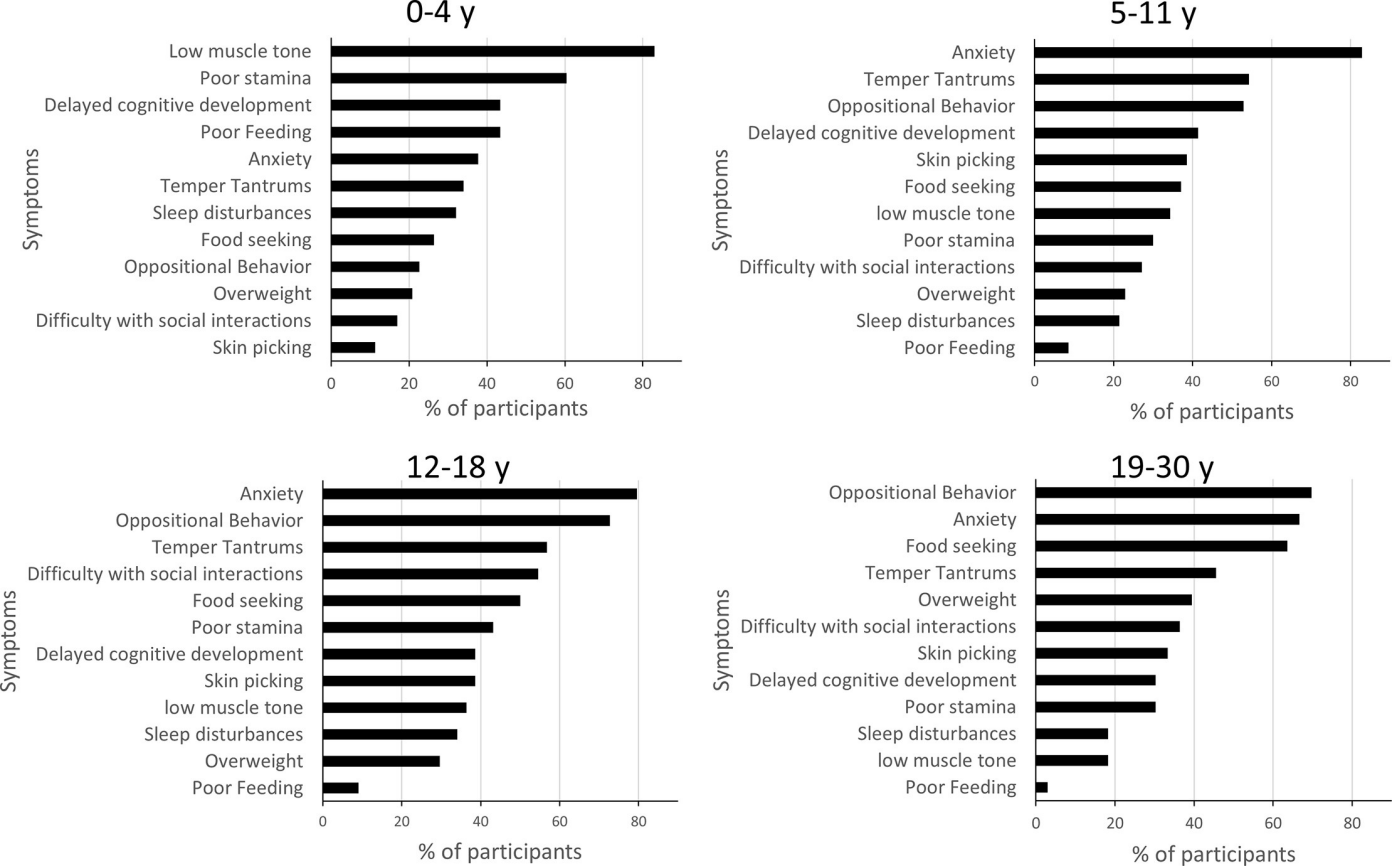
PWS symptoms that impact the person with PWS across age groups. Caregivers were asked to choose 3 symptoms amongst a list of 12 that had the biggest negative impact on the person with PWS. Results are expressed as the % of participants who selected a given symptom. Data for the 31 y and older age group were included into the statistical analyses but were not represented in this figure due to low number of participants (n = 4). Note that the proportion of participants who selected **poor feeding, **food seeking, **oppositional behavior, **anxiety, **poor stamina, **low muscle tone, *difficulty with social interactions and *skin picking was statistically different across age groups (* p < 0.05, ** p < 0.01).

Caregivers were asked to rank the 3 most impactful symptoms on individuals with PWS, selected from a list of 12 symptoms associated with PWS (Fig 4). Although the most impactful symptoms changed across age groups, anxiety (including repetitive questioning and obsessive-compulsive behavior) was rated as having the most negative impact on individuals with PWS for all ages combined (68% of caregivers). Anxiety’s negative impact increased with the age of individuals with PWS (p < 0.01) reaching the highest impact in individuals aged 5–30 y, and adults aged 31 y and older (75% of caregivers). Other behaviors that were rated as highly impactful by caregivers in the 5 y and above age groups were oppositional behavior (including arguing, inflexibility/rigidity) (52% of all caregivers) and temper tantrums (including meltdowns, poor emotional control, aggression) (48% of all caregivers). The impact of oppositional behavior increased with the individual’s age (p < 0.01), reaching its highest level in adolescents and young adults aged 12–30 y, and adults aged 31 y and older (75% of caregivers). Although temper tantrums did not change significantly across ages (p > 0.05), relative to other symptoms, they tended to have the greatest impact in children and adolescents aged 5–18 y. In contrast, low muscle tone (including hypotonia, delayed motor development) and poor stamina (including excessive sleepiness) were of particular concern in the 0–4 y age group, the impact of both symptoms decreasing as the individual’s age increased (p < 0.01). Food seeking (including excessive appetite, hyperphagia, overly focused on food) represented overall the fifth most important symptom (41% of caregivers). The impact of food seeking increased significantly with the age of individuals with PWS (p < 0.01), reaching its highest levels in individuals aged 19–30 y, but tended to decrease in adults aged 31 y and older (25% of caregivers).

Altogether, our results indicate that the most impactful symptoms changed across age groups. Symptoms with the biggest negative impact on individuals aged 0–4 y were low muscle tone, poor stamina, delayed cognitive development and poor feeding. In contrast, anxiety, temper tantrums and oppositional behavior were the symptoms with the biggest impact on individuals aged 5–30 y whereas food seeking had a preeminent impact in young adults with PWS aged 19–30 y.

### PWS symptoms and care-related issues that have the biggest negative impact on caregivers

**Fig 5:**
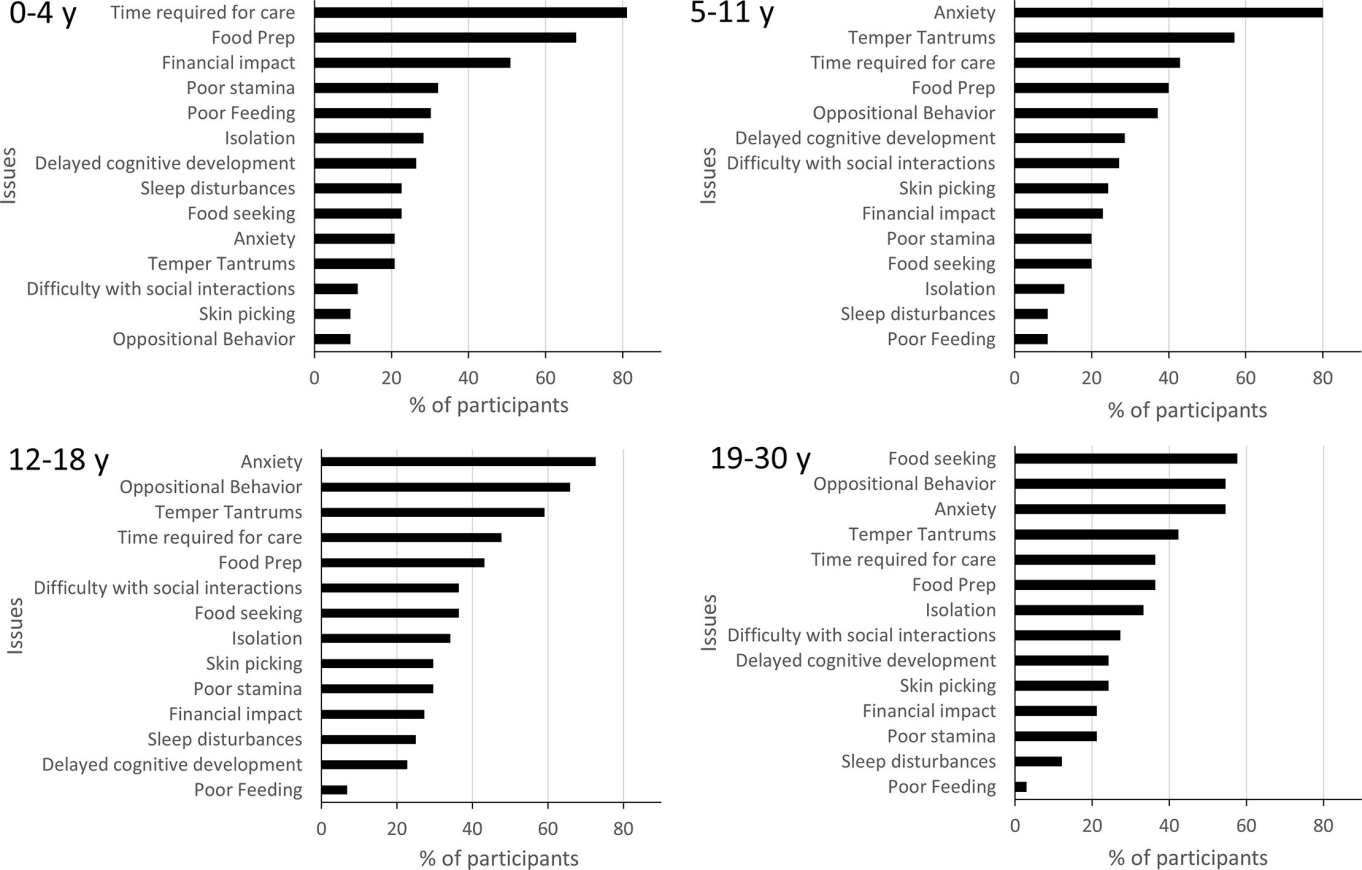
PWS issues that impact caregivers across age groups. Caregivers were asked to choose 3 issues amongst a list of 14 PWS symptoms and care related issues that had the biggest negative impact on them. Results are expressed as the % of participants who selected a given issue. Data for the 31 y and older age group were not represented in this figure due to low number of participants (n = 4). Note that the proportion of participants who selected **time required for care, **financial impact, **poor feeding, **food prep, **food seeking, *temper tantrums, **anxiety, **oppositional behavior, *difficulty with social interactions, was statistically different across age groups (* p < 0.05, ** p < 0.01).

Caregivers were next asked to choose 3 issues amongst a list of 14 PWS symptoms and care-related issues that had the greatest impact on them (Fig 5). The anxiety of the person with PWS was the most important issue for caregivers of individuals of all ages combined (59%). The negative impact of anxiety on caregivers increased as age of the person with PWS increased (p < 0.01), reaching its highest impact in caregivers of individuals aged 5–30 y, and in older adults (75% caregivers). Time required for care and treatments (including doctor visits, therapy, assisting with things that a typical child/person would do independently), represented overall the second most important issue (52%), especially for caregivers of children aged 0–4 y (p < 0.01 when compared across ages). Food and diet preparation (including time spent planning menus, preparing specialized foods), was overall the third most important issue (47%), especially in caregivers of children aged 0–4 y, whereas its impact decreased with increased age of the individuals with PWS (p < 0.01). Temper tantrums and oppositional behavior by the person with PWS were important issues for 46% and 40% of all caregivers, respectively. The impact of temper tantrums on caregivers increased as the age of individuals with PWS increased (p < 0.05) reaching its highest impact in caregivers of individuals aged 5 y and above. Likewise, the impact of oppositional behavior on caregivers increased with the age of individuals with PWS (p < 0.01). However, compared to temper tantrums, the highest impact of oppositional behavior was slightly shifted towards caregivers of adolescents and adults including older adults (100%). Although only 30% of all caregivers selected financial impact (including costs of therapy, medications, extra paid caregivers) as an important issue, it represented an important issue in caregivers of children aged 0–4 y (p < 0.01 when compared across ages). Of interest, food seeking by the person with PWS was rated as one of the most important issue in only 30% of caregivers of individuals of all ages combined. The negative impact of food seeking on caregivers increased, however, with the age of individuals with PWS (p < 0.01), and was the most important issue in caregivers of adults aged 19–30 y.

Altogether, our results showed that the 3 issues with the biggest negative impact on caregivers vary with the age of the individuals with PWS. They were: time required for care, food preparation and financial impact for caregivers of children aged 0–4 y; anxiety, temper tantrums and time required for care for caregivers of individuals aged 5–11 y; anxiety, oppositional behavior and temper tantrums for caregivers of individuals aged 12 y and older in addition to food seeking for the 19–30 y group.

### Stability of HQ-CT and ZBI scores over 6 months

Out of the 204 study participants, 83 participants (41%) responded to the second survey 6 months after completing the initial survey. The characteristics of caregivers and individuals with PWS who took part in the second survey were comparable to that of the larger sample (S1 Table), except for a slightly over-representation of caregivers with an annual household income equal or greater than $100,000 (+ 21%) in this subset when compared to the larger sample.

We next looked at significant life events or changes that may have occurred in the last 6 months in the lives of individuals with PWS and their caregivers. Overall, 77% of caregivers reported no changes in their lives whereas 45% of individuals with PWS had a significant life change, including treatment for mood or behavior (26%), change in living situation (8%), health (5%), treatment for hunger (4%) or other significant changes (10%).

#### HQ-CT scores over 6 months

**Fig 6:**
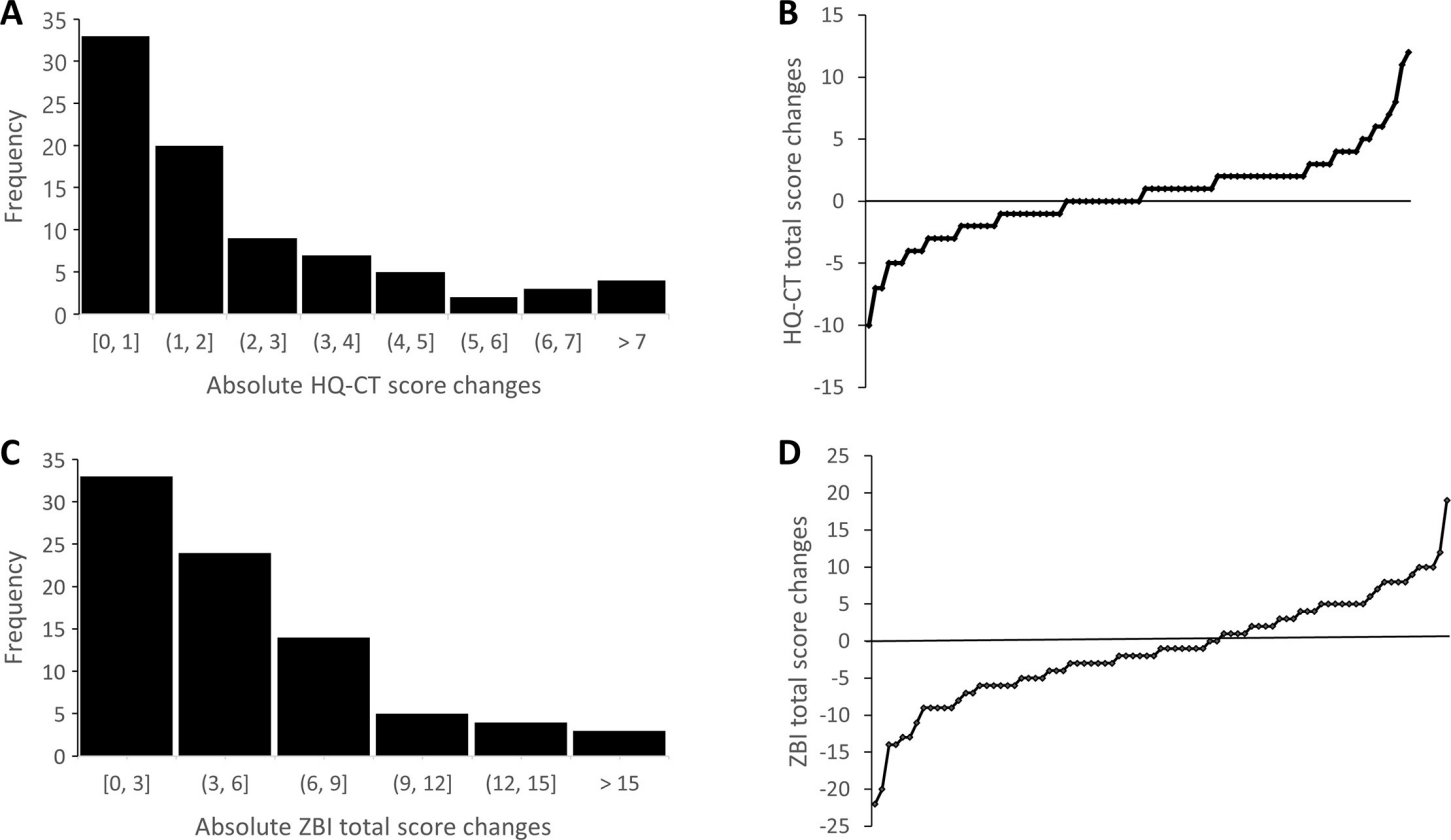
**Global changes over 6 months of individual HQ-CT (upper panel) and ZBI total score (lower panel).** Frequency distribution of absolute changes in 6 months of (A) HQ-CT total score with 1-unit increment and (C) ZBI total score with 3 points increment. B and D. Waterfall plot of (B) HQ-CT total score and (D) ZBI total score changes in 6 months.

The mean (SD) 6-month HQ-CT score change from baseline for the 83 individuals with PWS was 0.47 (3.58) with a median score change of 0 and score changes ranging from -10 to 12. There was no significant difference in the scores between 6 months and baseline (t (82) = 1.20, p > 0.1). When looking at individual’s absolute 6-month HQ-CT score changes from baseline (Fig 6A), most individuals (64%) had a relatively small score change ranging between 0 and 2 units, 25% had a change of 1 unit, 12% had no score changes and less that 5% of individuals with PWS had a score change greater than 7 units. Nearly 50% of scores increased from baseline (positive score change values) while 36% of changes decreased (negative score change values) (Fig 6B).

**Fig 7:**
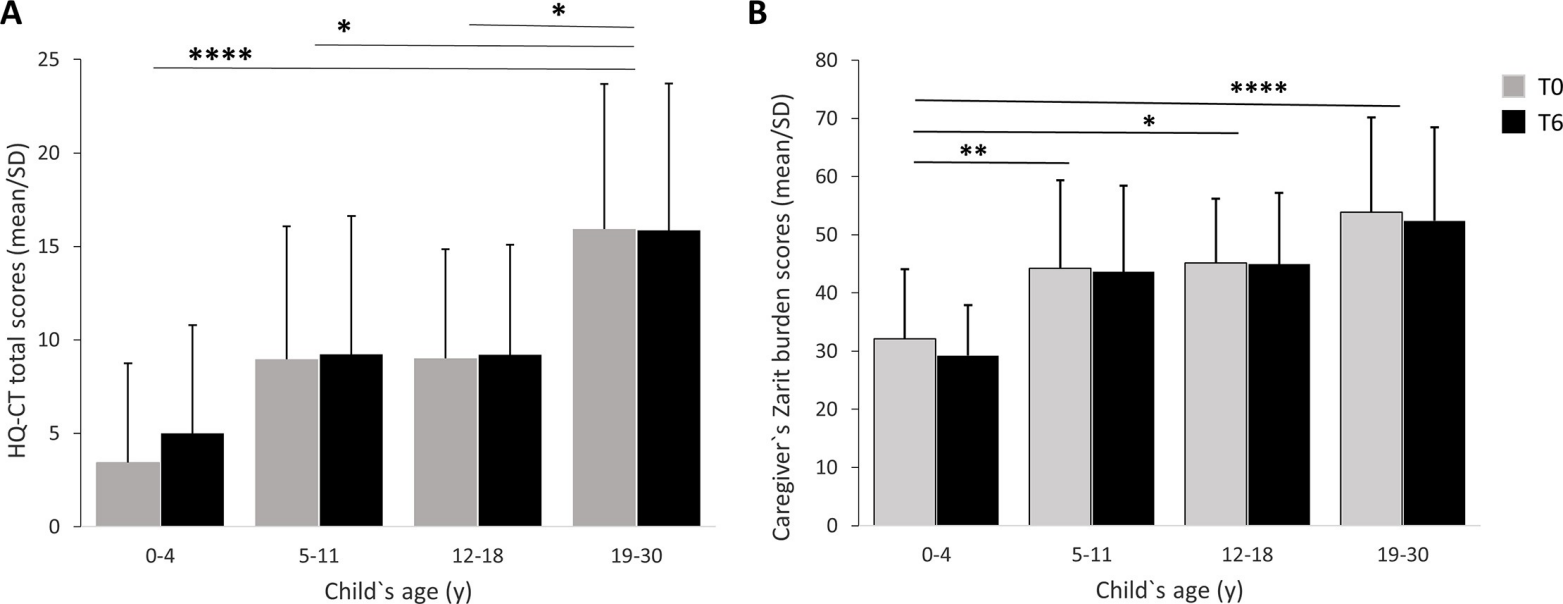
6-month variation of HQ-CT total score (A) and ZBI total score (B) as a function of the age of individuals with PWS. The 31 y and older age group which comprised only 4 individuals was included into the analysis but not represented in the figure. (A) HQ-CT scores varied significantly across the 5-age groups of individuals with PWS with no score difference at baseline (T0) and at 6 months (T6). *p < 0.05, ****p < 0.0001 when compared to 19–30 y category. (B) ZBI scores varied significantly across the 5-age groups of individuals with PWS with no score difference at baseline (T0) and at 6 months (T6). *p < 0.05, **p < 0.005, ****p < 0.0001 when compared to 0–4 y age category.

We found that HQ-CT scores varied significantly across the 5-age groups of individuals with PWS (F (4, 78) = 7.65, p < 0.0001) (Fig 7A) with no significant difference of the scores at baseline and at 6 months (F (1,78) = 1.43, p > 0.1), and no statistically significant interaction between individual’s age group and time of survey (F (4, 78) = 1.02, p > 0.1). Comparison between age groups, using Tukey’s adjustments, showed a statistically significant difference for the 19–30 y group when compared to the 0–4 y (p < 0.0001), 5–11 y (p < 0.05) and 12–18 y (p < 0.05) age groups. There was no statistically significant difference observed among all the other age groups including individuals aged 31 y and older (p > 0.05), albeit a trend toward statistically significant difference between the 0–4 y and 5–11 y age groups (p = 0.0538).

We next tested whether changes that occurred in the lives of caregivers and/or individuals with PWS in the past 6 months had an impact on HQ-CT scores. We found that changes in the lives of individuals with PWS did not significantly impact their HQ-CT scores. The mean (SD) HQ-CT score change from baseline decreased by 0.19 (3.64) (median score change: -1) in individuals who had significant life changes whereas it increased by 1.00 (3.47) (median score change: +1) in those who had no changes in the last 6 months (t (81) = 1.52, p > 0.1). In contrast, we found that changes in the lives of caregivers had a small but statistically significant impact on the HQ-CT scores of their children. For caregivers who had a significant life event or change in the last 6 months, the mean (SD) HQ-CT score changes from baseline decreased by 1.26 (3.45) (median score change: -1) whereas it increased by 0.98 (3.48) (median score change: +1) in individuals whose caregivers had no changes in the last 6 months (t (81) = 2.48, p < 0.05).

Overall, these results show that HQ-CT scores are stable over 6 months independently of the age of individuals with PWS or changes that occurred in the lives of individuals with PWS. Changes in the caregiver’s life had a statistically significant impact albeit very small (1 point) on HQ-CT scores.

#### ZBI scores over 6 months

For the 83 caregivers, the mean (SD) ZBI score change from baseline was -1.19 (7.10) with a median score change of -1 with score changes ranging from -22 to 19. There was no significant difference between the scores at 6 months and baseline (t (82) = -1.53, p > 0.1). When looking at individual’s absolute ZBI changes from baseline (Fig 6C), most caregivers (69%) had a relatively small score change ranging from 0 to 6 points including 40% with a change of 3 points or less. Nearly 60% of scores at 6 months decreased from baseline while nearly 40% scores increased from baseline (Fig 6D).

ZBI scores varied significantly across the 5-age groups of individuals with PWS (F (4,78) = 7.40, p < 0.0001) (Fig 7B) with no significant difference in the scores at baseline and at 6 months (F (1,78) = 2.34, p > 0.1) and no statistically significant interaction between age groups and time of the survey (F (4,78) = 0.62, p > 0.5). Comparison between age groups, using Tukey’s adjustments, showed a statistically significant difference for caregivers of children aged 0–4 y when compared to caregivers of children aged 5–11 y (p < 0.005), 12–18 y (p < 0.05), and 19–30 y (p < 0.0001). There was no statistically significant difference observed among the other age groups including individuals aged 31 y and older (p > 0.1).

We next tested the impact of life change or event on ZBI scores. We found that changes in the lives of individuals with PWS or their caregivers did not significantly impact caregiver ZBI scores. The mean (SD) ZBI score change from baseline decreased by 1.51 (6.56) (median score change: -1) in caregivers of individuals who had significant life changes and decreased by 0.94 (7.58) (median score change: -2) in those who had no life changes in the last 6 months (t (81) = 0.37, p > 0.5). The mean (SD) ZBI score change from baseline decreased by 3.79 (6.72) (median score change: -3) in caregivers who had a life change whereas it decreased by 0.42 (7.08) (median score change: -1) in caregivers who had no life change in the last 6 months (t (81) = 1.84, p > 0.05).

Overall, these results showed that ZBI scores are stable over a 6-month interval independently of the age of individuals with PWS or changes that occurred in the lives of individuals with PWS. Changes in the caregiver’s life had a modest but not statistically significant impact on ZBI scores (-3 points).

## Discussion

The main aims of the study were to assess the distribution of HQ-CT scores across PWS age groups, determine the stability of HQ-CT and ZBI scores over a 6-month period, examine the relationship between HQ-CT and ZBI, and to explore the contribution of other factors to caregiver burden in PWS. We found high levels of caregiver burden in PWS with ZBI scores comparable to those found in our previous study [16]. Similarly, we also found that the levels of caregiver burden increased as the individual with PWS ages. The results showed that the degree of hyperphagia and the level of caregiver burden varied concomitantly and significantly with the age of individuals with PWS. Further, the degree of hyperphagia is associated with the level of caregiver burden. The level of caregiver burden and degree of hyperphagia appeared to be independent of the weight status of individuals with PWS, except for those who were obese. To explore the contribution of other factors to caregiver burden, we looked at the negative impact of PWS symptoms and issues that affected caregivers the most. Interestingly, we found that they varied significantly with the age of individuals with PWS. Whereas tasks associated with caring for a child with PWS had the most negative impact on caregivers of infants, the burden of caregivers of children, adolescent and young adults was associated predominantly with the child’s anxiety and behavioral challenges. Finally, we showed that HQ-CT and ZBI measures were stable over a 6 month-period. Altogether, these new findings shed light on factors underlying caregiver burden in PWS and support ZBI as an additional outcome measure in clinical trials for PWS.

The present study brings three lines of evidence to support hyperphagia as a major contributor to caregiver burden in PWS. First, we showed high temporal similarity between the profiles of HQ-CT and ZBI scores (Fig 1), with both scores tended to increase with the child’s age. Second, we found a strong correlation between ZBI and HQ-CT scores in individuals with PWS above 4 years old. Third, we showed that HQ-CT scores demonstrated a strong relationship with ZBI scores. These results are not surprising given the life-threatening nature of hyperphagia and the 24/7 management and supervision measures that caregivers and families have to put in place to ensure food security for individuals with PWS. To our knowledge, this is the first study to provide direct evidence of a relationship between caregiver burden and hyperphagia in PWS.

Early diagnosis in PWS has allowed early dietary regimens and food security measures to control weight gain and prevent obesity-related morbidity [32]. However, these measures have little or no effect on hyperphagia [33]. Here, we provide evidence that, overall, the weight status of individuals with PWS is not predictive of the level of hyperphagia of individuals with PWS, except for obese individuals who had significantly higher degree of hyperphagia when compared to normal weight PWS individuals in agreement with another study using a different questionnaire [29]. We also showed that the level of burden in caregivers is independent of their child’s weight status. Altogether, our results extend previous findings and suggest a clear distinction between hyperphagia and weight in terms of stress impact on families [12], treatment priorities [34], and caregiver burden (this study).

To identify other factors that may contribute to caregiver burden, we measured the negative impact of PWS symptoms and care-related issues on individuals with PWS and caregivers. We found that the impact on individuals and caregivers changed across age groups, likely reflecting the natural history of the disorder. Interestingly, we found a clear difference between caregivers of children younger or older than 4 years old. Caregivers of children above 4 y, adolescents, and young adults were predominantly impacted by the PWS symptoms that specifically affect individuals in this age range, namely anxiety, temper tantrums and oppositional behavior. Behavioral challenges have been shown to emerge around 4 y [2, 35, 36], in agreement with this study. Greater behavioral problems are seen as the person with PWS ages, especially during adolescence and young adulthood [3, 5, 24–27]. In contrast, there have been mixed results on anxiety in PWS. Some studies in PWS did not appear to capture the challenge of anxiety in the PWS population [37, 38]. Other studies have, however, found moderate to high levels of anxiety in PWS samples [3, 7, 39]. Different approaches in assessing anxiety were utilized in these studies that may explain the discrepancies between results. The lack of validated outcome measures specific for anxiety, temper tantrums and oppositional behaviors in PWS prevented direct assessment of the relationship between these symptoms and caregiver burden. Nevertheless, the timing of the impact of these symptoms and the concomitant and significant elevated ZBI scores found in these age categories suggests that anxiety and behavioral challenges probably account for a significant portion of the burden of caregivers of individuals with PWS above 4 y and may explain the results showing that caregiver burden in PWS tends to increase as the individual with PWS ages [16] (this paper). It is noteworthy that despite hyperphagia being the hallmark symptom for PWS, a symptom reflective of this issue (i.e. food seeking) was consistently ranked lower than anxiety and behavioral challenges with respect to its impact on the person with PWS and on the caregiver in the 5–18 y age groups, whereas it ranked higher in caregivers of young adults aged 19–30 y. This is consistent with the findings that HQ-CT scores increased with age and were highest in the 19–30 y age category. These results also suggest that anxiety and behavioral challenges may contribute significantly to the burden of caregivers of children and adolescents in the 5–18 y age category, while hyperphagia may contribute the most to the burden of caregivers of young adults in the 19–30 y category. In a recent publication examining caregiver preferences for PWS treatment, caregivers rated hyperphagia and anxiety as their top two targets for PWS treatment [34]. Additionally, the results of a recent survey found that “difficult behavior, not food related” was a top-rated issue impacting the person with PWS in their day-to-day life [28]. In contrast, the burden of caregivers of infants and young children aged 0–4 y was associated primarily with time required for care, food preparation and financial impact. This may reflect the time intensive demands of health care and therapies common for youngest child with PWS and the likely taxing effect as caregivers adjust to their “new normal”.

HQ-CT has good validity and reliability and is currently the gold standard outcome measure in clinical trials for hyperphagia in PWS [40, 41]. Until this study, there were no data on the variability of HQ-CT scores across lifespan and its stability overtime, both of which are important when assessing efficacy of a treatment against hyperphagia. Here, we showed that HQ-CT scores significantly differentiated infants and young children aged 0–4 y from older individuals with PWS. The majority of infants and children had low HQ-CT scores (below 4 units) likely reflecting the poor feeding that was found to have a high negative impact on children in this age group (Fig 4). In contrast, HQ-CT scores were significantly higher in individuals 5 years and above (scores ≥ 10 units), tended to increase with the age of individuals concomitant to the increased negative impact of food seeking on these individuals and their caregivers. These results suggest that HQ-CT scores reflect the typical gradual progression of food-related behaviors in PWS beginning with feeding difficulty in infancy moving to an increase interest in food and finally to severe hyperphagia [33]. Few studies of HQ-CT data have been published which limits comparison of our results with others. Two recent reports of clinical trials for hyperphagia have reported baseline mean HQ-CT scores between 15 and 18.3 in obese adolescents and adults with PWS [41, 42], scores that are much higher than the mean score (12.2) we found in obese individuals in our study. Another recent clinical study also reported higher baseline mean HQ-CT scores (around 18) in children with PWS aged 3–11 y [43]. The difference of research settings may explain the higher baseline scores in these clinical studies compared to the current study. The inclusion/exclusion criteria of clinical trials may select a specific subset of the PWS population. One example is the bestPWS trial which selected obese participants with HQ-CT scores above 13 [41]. Another bias could be that families who choose to participate in hyperphagia-related clinical studies may represent individuals with PWS with the most severe degree of hyperphagia. Nonetheless, our study provides the first reference HQ-CT dataset in real-world conditions providing valuable information about the natural history of hyperphagia in a large sample of individuals with PWS.

Therapeutic decisions demand reliable outcome measures to ensure that an observed change in that measure reflects changes of the disease activity rather than the natural variation of the measure itself. ZBI and HQ-CT measures have previously shown good reliability, consistency and validity. In the present study, we examined the natural variation of HQ-CT and ZBI over 6 months, which is often the duration of a phase 3 clinical trial in PWS population. Data was also collected on the changes that occurred in the lives of caregivers or their children with PWS to distinguish changes in the measures caused by life events from those due to the natural variation of the measures. We found that HQ-CT scores were overall stable over 6 months, with a natural variation below two units for most participants. In addition, changes in the individual or caregiver’s life did not meaningfully change HQ-CT scores (less than 1 unit). These results are not surprising given the lack of treatment for hyperphagia and suggests that HQ-CT is a reliable caregiver reported measure of food related behaviors of individuals with PWS. Likewise, we found that ZBI scores were stable, with a small natural variation below 6 points over 6 months, independent of changes that occurred in the lives of caregivers or individuals with PWS. Our findings should help to interpret these measures, specifically their sensitivity to intervention-induced changes over time in the context of clinical trials and clinical research and to power future research studies.

Interventional studies to reduce caregiver burden across all types of chronic diseases have mostly consisted of developing support interventions or testing pharmacological agents to reduce symptoms directly in caregivers [44, 45]. The impact on caregiver burden of therapeutic interventions that focus on improving patient symptoms has rarely been tested. Our study showed a direct relationship between hyperphagia and caregiver burden and provided some indirect evidence for a relation between anxiety, behavioral challenges and caregiver burden in PWS. These results open up the possibility for future clinical trials to incorporate ZBI as an observer-reported outcome measure to test whether therapies targeting the patient`s hyperphagia, anxiety or behavioral challenges can also improve caregiver burden, in turn decreasing healthcare costs associated with PWS [46, 47].

While this study has important implications for the PWS population and clinical research, several limitations should be noted. The cross-sectional design suggests caution in the extrapolation of the findings regarding directionality or causation. The majority of study participants were mothers, married, Caucasian, between 30 and 59 years of age, and with a higher income, all of which could call into question the generalizability of our findings. The lack of ethnic or racial difference in PWS prevalence points towards a bias in web-based survey participation or online recruitment strategies that may disproportionally affect different groups of race or ethnicity [48]. The results of this study are also mostly applicable to individuals with PWS living at home. Attrition of older participant limited our ability to study older adults with PWS. Another limitation was the lack of validated measures of anxiety and behavioral challenges in PWS that prevented direct assessment of the relationship between these symptoms and caregiver burden.

## Conclusions

Outside of its limitations, the present study provides for the first time a dataset of the natural variation of HQ-CT and ZBI over age and time and gives insight into the factors contributing to caregiver burden in PWS opening the path for incorporating ZBI in future clinical trials for PWS.

## Supporting information

S1 Appendix Assessment of the impact of PWS symptoms and caregiver-related issues. (DOCX)

S1 Table Sociodemographic characteristics of the caregivers and individuals with PWS who participated in the 6 months study. (DOCX)
