# Supplementary material for: Characteristics and relationship between hyperphagia, anxiety, behavioral challenges and caregiver burden in Prader-Willi syndrome
Source: PLoS One. 2021 Mar 25;16(3):e0248739. doi: 10.1371/journal.pone.0248739 (PMC7993772; doi:10.1371/journal.pone.0248739)
Supplement: S1 Appendix — (DOCX) [file pone.0248739.s001.docx]

**Impact of PWS Symptoms on the Person with PWS**

Of the following list of symptoms associated with PWS, choose the **3** symptoms that have the biggest negative impact on the person with PWS. For these top **3** symptoms, please indicate how challenging they are, on a scale where 0= not at all a challenge and 9 = extreme challenge (Choose only the Top 3 to rate)

1. Poor feeding (includes low appetite, difficulty sucking/swallowing)

2. Food seeking (includes excessive appetite, hyperphagia, overly focused food )

3. Temper tantrums (includes meltdowns, poor emotional control, aggression)

4. Anxiety (includes repetitive questioning, obsessive compulsive behavior)

5. Oppositional behavior (includes arguing, inflexibility/rigidity)

6. Difficulty with social interaction (poor social/friendship skills)

7. Overweight (includes extra weight, obesity, easy weight gain)

8. Poor stamina/low energy (includes excessive sleepiness)

9. Skin picking (includes nail picking, pulling hair out)

10. Low muscle tone /hypotonia (includes delayed motor development)

11. Delayed cognitive development / intellectual disability

12. Sleep disturbances /sleep apnea

**Impact of PWS Symptoms on the Caregiver -**

Of the following issues associated with PWS, choose the **3** that have the greatest impact on *you*.

Indicate the level of severity of these Top 3 issues for you on a scale from 0 (not a challenge) to 9 (extremely challenging)

1. Poor feeding (includes low appetite, difficulty sucking/swallowing)
2. Diet and food preparation (includes time spent planning menus, preparing specialized foods)
3. Food seeking by the person with PWS (includes excessive appetite, hyperphagia, overly focused food)
4. Temper tantrums by the person with PWS (includes meltdowns, poor emotional control, aggression)
5. Anxiety of the person with PWS (includes repetitive questioning, obsessive compulsive behavior)
6. Oppositional behavior by the person with PWS (includes arguing, inflexibility/rigidity)
7. Difficulty with social interaction (poor social/friendship skills)
8. Poor stamina/low energy (includes excessive sleepiness)
9. Skin picking (includes nail picking, pulling hair out)
10. Time required for care /treatments (includes doctor visits, therapy, assisting with things that a typical child/person would do independently)
11. Delayed cognitive development / intellectual disability
12. Sleep disturbances /sleep apnea
13. Isolation (includes social isolation, lack of local support)
14. Financial impact (costs of therapy, medications, extra paid caregivers).
