# Supplementary material for: Characteristics and relationship between hyperphagia, anxiety, behavioral challenges and caregiver burden in Prader-Willi syndrome
Source: PLoS One. 2021 Mar 25;16(3):e0248739. doi: 10.1371/journal.pone.0248739 (PMC7993772; doi:10.1371/journal.pone.0248739)
Supplement: Supplementary file 2 [file pone.0248739.s002.docx]

Variable	N (%)	Variable	N (%)
**Caregivers**			
Relationship with Child		Annual household income	
Parent	82 (98.8)	Less than $10000	2 (2.41)
Grand-parent	0	$10000 - $14999	3 (3.61)
Sibling	0	$15000 - $19999	1 (1.2)
Legal guardian	1 (1.2)	$20000 - $24999	1 (1.2)
Age (years)		$25000 - $29999	0
20-29	5 (6.02)	$30000 - $34999	2 (2.41)
30-39	25 (30.12)	$35000 - $39999	1 (1.2)
40-49	30 (36.14)	$40000 - $44999	3 (3.61)
50-59	20 (24.1)	$45000 - $49999	1 (1.2)
60-69	2 (2.41)	$50000 - $54999	2 (2.41)
70-79	1 (1.2)	$55000 - $59999	1 (1.2)
Marital status		$60000 - $74999	7 (8.4)
Married	75 (90.36)	$75000 - $84999	5 (6.02)
Living with partner	0	$85000 - $99999	0
Divorced	4 (4.82)	$100000 - $149999	33 (39.76)
Separated	2 (2.41)	$150000 - $199999	6 (7.23)
Widow	0	$200000 - $249999	5 (6.02)
Single	2 (2.41)	$250000 and above	8 (9.64)
Ethnicity		Don't know	2 (2.41)
Asian	1 (1.2)	**Individuals with PWS**	
Caucasian/ White non-Hispanic	77 (92.77)	Age (years)	
Black / African American	1 (1.2)	0-4	21 (25.3)
Hispanic/ Latino	1 (1.2)	5-11	31 (37.35)
American Indian/Alaska Native	0	12-18	15 (18.07)
Multi-ethnic	2 (2.41)	19-30	15 (18.07)
Other	0	31+	1 (1.20)
Decline to answer	1 (1.2)	Living situations	
Household		One or both parents	80 (96.39)
2	4 (4.82)	Residential/Boarding school	0
3	21 (25.3)	Independent apartment/house with supports	1 (1.2)
4	31 (37.35)	Small supported living (4 or less)	0
5	19 (22.89)	Group home (5 or more)	1 (1.2)
6	6 (7.23)	Other	0
7	1 (1.2)	Time living with caregivers	
8	1 (1.2)	0-25%	2 (2.41)
		26-50%	1 (1.2)
		51-75%	2 (2.41)
		76-100%	78 (93.98)
